# The Early Evolution of Surgery

**Published:** 1920-01-10

**Authors:** 


### THE EARLY EVOLUTION OF SURGERY.

Although the latter-day developments of surgery are by far the most important, and probably the more familiar to us, yet the earlier traditions upon which so much time and labour were spent by the ancients and the savants of the Middle Ages are not without interest in the light of recent events in the surgical world. Throughout the ages war has had a most devastating effect on contemporary surgical procedure, and the Great War has been no exception to this rule, the guillotine amputation of A.D. 1917 being no less drastic than the "Primary Amputation" of Paré in 1552.

The recently instituted "Thomas Vicary Lecture" delivered by Sir Thomas Tweedy, LL.D., F.R.C.S., before the Royal College of Surgeons, deals with this subject in a masterly manner, and gives us a most interesting and co-ordinate narration, collaborating so many of those historic innovations which, interesting enough in themselves, lose much of their value unless arranged in orderly sequence

### Homer and Herodotus.

Even from prehistoric times we possess evidences of the practice of operative surgery, such evidence being, of course, osseous and furnished by the remains of skulls and bones showing marks of surgical treatment, the soft parts having perished. All are familiar with Homer's praise of the surgeons in the Trojan war, more particularly Podalirius and Machaon, whose effigies are represented as the supporters in the heraldic arms of the College of Surgeons of England. In several places Homer describes injuries received in battle, and his descriptions of the effects of these injuries manifest a notable acquaintance with anatomy and physiology and with the effects of loss of blood and of shock. Herodotus records many events of medical and surgical interest, especially in Persia and ancient Egypt; in the latter country medicine and surgery early attained a high standard of proficiency, but they soon split up into a large number of specialities. Eventually extreme specialism put a stop to progress. Every practitioner was compelled to follow the rules for the treatment of disease as prescribed by the sacred books, and departure from these rules was a capital offence if the patient died.

### The Surgery of Hippocrates.

The works of this great man, although usually associated with medicine, have great practical surgical value; in fact, some of his principles have lasted right up to the present time. The Hippocratic treatises on wounds and ulcers and on injuries of the head are replete with well-observed facts, and are full of excellent teaching concerning the treatment of wounds, and especially the extreme importance of cleanliness in dressings and instruments. Thus a bridge of more than two thousand years rests between the initiation of aseptic surgery and its fruition in the Listerian era. Littré is said to have described the treatises on fractures and dislocations as "the grandest surgical monument of antiquity."

### Celsus and the Early Christian Era.

Four hundred years after the days of Hippocrates we come to the writings of Celsus, who in the sixth and seventh volumes of his famous "De Medicina" compiles most of the surgical achievements and progress in the Middle Ages. As Sir John Tweedy remarks, "Very little is known of Celsus's personality"; he was regarded by Professor Broca rather as a book or an epoch than as a man. But for Celsus we should know little of the famous school of Alexandria, where, under the inspiration of Erasistratus and Herophilus and their disciples, anatomy and surgery were more studied than ever before, and where surgery made more progress in 300 years than in the following thirteen centuries. Amongst the various operative procedures advocated by this author, those of ligation for bleeding arteries are mentioned; the complete division of the vessel (after ligation both above and below the site of injury) is advised.

For many years after the death of Celsus his writings, for some unknown reason, seem to have been entirely forgotten until the fifteenth century, when a copy of his "De Medicina" was found in the year 1443 by Thomas of Sazanne, afterwards Pope Nicholas V., in the church of St. Ambrose of Milan. Celsus soon had his revenge, for on the invention of the "Printing Press" his was one of the first medical writings to be issued. The first edition was printed in the year A.D. 1478, five years before the Ars Parva of Galen and the Aphorisms of Hippocrates.

Later, with Paulus Aegineta, closes the era of classical Greek medicine and its traditions, which would probably have been completely extinguished but for the intervention of the Mohammedans, who in the seventh and eighth centuries overran and conquered Syria, Persia, and Egypt.

### Arabian Surgery.

Strictly speaking, there is no Arabian science of surgery; there is Greek, Persian, Spanish, and Jewish. The Arabians had no taste or training for scientific or philosophical pursuits, but in the countries which they conquered they came in contact with much Greek and Oriental learning, which they soon observed and assimilated. Although the Mohammedans at first manifested fanatical hostility against much of the Greek learning, they made an exception in favour of medicine.

In Persia and in Asia Minor there were many Greek philosophers and physicians, not a few of whom were Christians, especially the Nestorians. Many Nestorian Syrians lived among the Arabians as physicians. Hareth ibn Kalda, the friend and physician of Mohammed himself, was a Nestorian. Schools of translators were established, the most famous of which was situate at Baghdad, the head of the school being the Nestorian Honain ibn Ishak. The most famous exponent of Arabian surgery, thus called, was Albucasis of Cordova, who practised in Spain about the end of the eleventh century. It is probable, however, that we can date the revival of surgery in mediæval times from the translation of Albucasis' works into Latin by Gerard of Cremona in Italy.

Many Italian medical practitioners left their country in consequence of the troubles caused by the quarrels of the Guelphs and the Ghibellines and sought refuge on French soil. One of the first of these migrants was Roger, of Parma; following him, Brudel, of Calabria, Lanfranchi, and others. Lanfranchi delivered a course of lectures on surgery in Paris about the year 1295. These were the first systematic lectures on surgery delivered in Paris. The influence of Lanfranchi in France was very transitory, notwithstanding the endeavours of Henri of Mandeville, who, early in the fourteenth century, wrote the first work on surgery in the French language. Henri of Mandeville was soon eclipsed by Guy, whose book is one of the best ever written. Even to-day it may be perused by anyone interested in the surgery of the Middle Ages.

**The Early Evolution of Surgery**—(*continued*).

In his remarkable introductory chapter he defines the nature and object of surgery. It is, he says, the science which teaches the manner and quality of operating, principally in consolidating, in seizing and performing other manual operations, and healing men so far as it is possible. In the same chapter he traces briefly the history of surgery from the time of Hippocrates downwards, and remarks that up to the time of Albucasis the medical writers had all been both physicians and surgeons, that after him surgery became separated from medicine, and fell into the hands of mechanics. And here I would emphasise the important fact that in studying the history of surgery it should never be forgotten that Hippocrates and all the classical Greek and Mohammedan authors considered surgery as an inseparable part of medicine. Surgery, as defined by Celsus, is "that branch of therapeutics which treats by manual operations, the other two branches being, first, that which treats by *régime* or dietetics, and, second, that which treats by pharmaceutics." "None of these is independent or autonomous, but, in their totality, constitute the whole of the medical art." Guy's idea of a surgeon was that he should be expert and well-mannered, bold when sure, cautious among dangers. Personally, Guy had great moral courage, as is shown by his determination to remain at Avignon during the "Black Visitation" in 1348. He was himself attacked, but recovered, and was at the post of professional duty at the time of the next visitation of the Plague in the year 1360.

As a practical surgeon, Guy sometimes erred on the side of over-caution. In his account of the treatment of gangrenous limbs he describes the operation of amputation as it is given in Paulus, but concludes by remarking: "As for myself, in these cases I envelop the whole of the mortified limb with many layers of adhesive plaster, and then tie it up, maintaining the constriction until the line of demarcation liquefies and the limb drops off of itself." It brings more credit to the surgeon, he remarks, to let the limb drop off than to cut it off, because when it is cut there is always a feeling of rancour or regret in the patient's mind that the limb might have remained.

Guy was almost the only authority on surgery for at least two centuries—that is, until the time of Ambrose Paré, born in 1517, who went to Paris and became apprenticed to a barber-surgeon. Malgine, in his learned introduction to Paré's work, states it is almost certain that the principal, if not the only, books read in Paré's early days were those of Guy and a French translation of Vigo. As showing the esteem in which these works were held, he remarks that Vicary bequeathed his copy of Guy's "Surgery" to the Barbers' Company, and his copy of "John of Vigo" to one of his assistants. Guy was the more learned, but Paré was unquestionably the better and bolder surgeon. He was not hampered by the trammels of authority. His experience was eminently practical, and in his earlier life was acquired almost exclusively in the wars.

Although firearms had been used in battle for a couple of centuries or more before Paré's time, it was only at the end of the fifteenth century that they became efficient weapons. The severity of the wounds, the smashing of bones, the laceration of flesh, and even the complete avulsion of limbs, the severe hæmorrhage and shock were then altogether different from anything the surgeons had previously known. These new experiences called for new procedures, and ultimately revolutionised the art of operative surgery. Up to Paré's time amputation of the limb was rarely performed, except for gangrene; but Paré had to face the imperative necessity of performing primary amputation. In amputation for gangrene, when the incision was made in or near the line of demarcation the hæmorrhage as a rule would be slight, and easily controlled by the actual or potential cautery, but in primary amputation it was soon found that the cautery was not sufficient. Even if the hæmorrhage were temporarily arrested, secondary hæmorrhage frequently followed. To avoid this danger, Paré proposed to tie the divided blood-vessels. There is some evidence that he did this for the first time in the year 1552, the year in which he was made Governor for life of St. Bartholomew's Hospital. And yet two and a half centuries more were to elapse before the true principles of arterial ligation were discovered. It must be remembered that Paré did not claim, as is sometimes thought, the credit of being the first to use ligatures for bleeding arteries. He knew that this was one of the modes for arresting hæmorrhage which had been known to and employed by surgeons for many centuries, but he was one of the first, if not the first, to employ the ligature in primary amputation. He did not know, however, the essential conditions for the successful application of the ligature. He was not careful to isolate the artery. The practice of tying an artery in amputation did not, therefore, commend itself to practical surgeons, and for many years was strongly opposed. Yet the idea was a compelling one. Even those who did employ the ligature were afraid to damage the coats of the vessels by too tight a constriction, and various contrivances were used to bind the vessel without injuring the coats. Even so sagacious a surgeon as John Hunter failed to discover the true path. In his first operation, the tying of the femoral artery for popliteal aneurism, he employed four ligatures, thinking thereby to ensure greater safety. Fortunately, the operation was successful, in spite of the risk incurred by using multiple ligatures.

Notwithstanding the failure and disappointment which attended the use of the ligature for more than two and a half centuries after Paré, observant practitioners were gradually making progress along two lines of advance. One set called attention to the increased safety of isolating the artery before tying it; the other demonstrated the greater safety of using fine ligatures tightly applied. But the full credit of discovering the true principles of arterial ligation belongs to Dr. J. F. D. Jones, a member of this college, who in 1805 published his famous treatise on the use of the ligature. By means of a few well-designed and careful experiments on animals, chiefly horses and dogs, Jones discovered that the only safe and sure method of tying an artery was to apply a small ligature with sufficient force to divide the inner and middle coats. Hundreds of surgeons must have done this without knowing it, but Jones demonstrated why and how it should be done in every case.

